# Outcomes of programmed death protein-1 inhibitors treatment of chronic active Epstein Barr virus infection: A single center retrospective analysis

**DOI:** 10.3389/fimmu.2023.1093719

**Published:** 2023-03-10

**Authors:** Yaxian Ma, Peiling Zhang, Yuhan Bao, Hui Luo, Jiachen Wang, Liang Huang, Miao Zheng

**Affiliations:** ^1^ Department of Hematology, Tongji Hospital, Tongji Medical College, Huazhong University of Science and Technology, Wuhan, China; ^2^ Immunotherapy Research Center for Hematologic Diseases of Hubei Province, Wuhan, China

**Keywords:** Epstein-Barr virus, chronic active Epstein-Barr virus infection, programmed death protein-1 inhibitors, lymphoma, immunotherapy

## Abstract

**Introduction:**

Chronic active Epstein-Barr virus (CAEBV) disease is a high-mortality disease, which is characterized by persistent infectious mononucleosis-like symptoms. There is no standard treatment for CAEBV and allogeneic hematopoietic stem cell transplantation (HSCT) was considered the only potentially therapeutic approach. PD-1 inhibitors have achieved high response in many Epstein-Barr virus-related diseases. In this single-center retrospective analysis, we report the outcomes of PD-1 inhibitors treatment of CAEBV.

**Methods:**

All CAEBV patients without hemophagocytic lymphohistiocytosis (HLH), who were treated with PD-1 inhibitors in our center between 6/1/2017 and 12/31/2021, were retrospectively analyzed. The efficacy and safety of the PD-1 inhibitors were evaluated.

**Results:**

Among the sixteen patients with a median age at onset of 33 years (range, 11-67 years), twelve patients responded to PD-1 inhibitors and the median progression-free survival (PFS) was 11.1 months (range, 4.9-54.8 months). Three achieved clinical complete response (clinical CR), as well as molecular CR. Five patients achieved and remained partial response (PR), and four converted from PR to no response (NR). For three CR patients, the median time and cycles from the first application of PD-1 inhibitor to clinical CR were 6 weeks (range, 4-10 weeks) and 3 cycles (range, 2-4 cycles), and molecular CR was achieved after a median of 16.7 weeks (range, 6.1-18.4 weeks) and 5 cycles (range, 3-6 cycles) of PD-1 inhibitor infusion. No immune-related adverse events have been observed except for one patient who suffered immune-related pancreatitis. There was no correlation of treatment outcome with blood count, liver function, LDH, cytokine or ferritin levels. NK cell function, PD-L1 expression in tumor tissue and gene mutation possibly correlated with treatment response.

**Discussion:**

In patients with CAEBV, PD-1 inhibitors have tolerable toxicity and comparable outcomes while improving quality of life and financial toxicity. Larger prospective studies and longer follow-up time is needed to be conducted.

## Introduction

Epstein–Barr virus (EBV), also known as human herpesvirus 4 (HHV-4), is a ubiquitous linear double-stranded DNA virus carried by approximately 95% of the adult population worldwide (1, 2). Primary infection occurs in adolescents or young adults and often results in infectious mononucleosis (IM), which typically manifests as fever, pharyngitis, lymphadenopathy, hepatosplenomegaly and atypical lymphocytosis (3, 4). In most cases, these manifestations of IM resolve spontaneously without sequelae after the emergence of EBV-specific immunity. Rare persons may develop a life-threatening syndrome called chronic active EBV infection (CAEBV), which is characterized by IM-like symptoms lasting for at least 3 months and high levels of blood EBV DNA in immunocompetent persons (5–7).

According to the most recent World Health Organization (WHO) classification of tumors of hematopoietic and lymphoid tissues, revised in 2017, CAEBV is now regarded as EBV-associated T or NK cell lymphoproliferative disorders (EBV+T/NK-LPDs) (7–9). The classification defines CAEBV into two subtypes: systemic CAEBV (sCAEBV), and cutaneous CAEBV of hypersensitivity to mosquito bite (HMB) or hydroa vacciniforme (HV) (8). In addition, some patients with CAEBV will develop hemophagocytic lymphohistiocytosis (HLH), lymphoproliferative disease (LPD) or lymphomas derived from T or NK cell lineages, which may lead to rapid disease progression and eventual death (10).

In terms of treatment, effective antiviral drugs and molecular targeted therapy for CAEBV have not yet been found. The disease is often resistant to chemotherapy and thus has a poor prognosis. Therefore, to date, the only proven therapy to effectively cure CAEBV is allogeneic hematopoietic stem cell transplantation (allo-HSCT) despite the high incidence of transplant-related complications (10–13). Additionally, the clinical application of allo-HSCT is limited by high cost and the availability of matched donors. So better treatment strategies are needed for CAEBV.

Programmed cell death protein-1 (PD-1) inhibitors have been approved for a variety of cancers (14). PD-1 and PD-1 ligand (PD-L1) are highly expressed in EBV-LPD pathological tissues (15–17), and PD-1 inhibitors have been shown to be effective against EBV+ gastric cancer (18) and refractory/relapse ENKTL (19–21). In addition, the study has shown that PD-1 inhibitors can achieve sustained control of refractory/relapse EBV-HLH with tolerable toxicity (22). Inhibition of the interaction between PD-1 and PD-L1 reverses EBV or cancer-related immunosuppression, which will restore the immunity needed to suppress cancer cells and clear EBV infection (14, 15). Therefore, we speculate that the PD-1 inhibitors regimen may be beneficial for CAEBV patients.

However, whether PD-1 inhibitors will be safe and effective for CAEBV has not been studied. Therefore, this study was conducted to evaluate the therapeutic effect and adverse effects of PD-1 inhibitors in CAEBV patients without HLH.

## Materials and methods

### Patient

We retrospectively analyzed the clinical data from 16 CAEBV patients treated with PD-1 inhibitors between 6/1/2017 and 12/31/2021. The diagnostic criteria for CAEBV include IM-like symptoms lasting for at least 3 months, elevated EBV DNA load in the peripheral blood (PB) or the tissue lesion, and demonstration of EBV infection in the affected tissues in patients without other possible diagnoses (IM, autoimmune diseases, malignancy, immunodeficiency, and other underlying diseases with potential immunosuppression) (7). HLH was excluded according to the HLH-2004 diagnosis guidelines (23). In addition, these Patients were tested for HIV and COVID-19 and there was no HIV-infected or COVID-19-infected in our cohort. There was no sign of co-infection with any other virus.

Patient characteristics, treatments and outcomes were collected, along with laboratory findings, including EBV-DNA copy number in PB and plasma, EBV sorting quantitative polymerase chain reaction (PCR), size of spleen and liver, blood count, levels of aspartate aminotransferase (AST) and alanine aminotransferase (ALT), fibrinogen, lactate dehydrogenase, cytokines and ferritin levels. This study was approved by the ethics committee of Tongji Medical College, Huazhong University of Science and Technology. Written informed consent was waived because of the retrospective nature of the study.

### Efficacy criteria

The outcomes of the treatment were evaluated as follows. Complete response (CR) was defined as the disappearance of all clinical symptoms, including fever, liver dysfunction, progressive skin lesions, or vasculitis (clinical CR) and the normalization of all abnormal laboratory indicators, including undetected EBV-DNA in plasma (molecular CR). Partial response (PR) was defined as resolution of some of the above symptoms of disease and laboratory parameters, including a 25% decrease in plasma EBV-DNA copy number, a 25% decrease in serum ferritin and triglyceride levels, a 100% increase in the blood neutrophil count or a 50% decrease in serum ALT levels in patients with liver dysfunction (22, 24). No response (NR) was defined as failure to meet the above criteria for CR and PR.

### EBV infection target cell type detection

Ficoll was used to separate peripheral blood mononuclear cells (PBMC) from peripheral blood samples, and then immunomagnetic beads were used to sort T cells (CD3+, Miltenyi Biotec), B cells (CD19+, Miltenyi Biotec) and NK cells (NK Cell Isolation Kit, Miltenyi Biotec). The EBV expression levels were quantified by real-time quantitative PCR (qPCR) in purified and amplified T, B, NK and PBMC cells. Taking the EBV-DNA copy number in PBMC as the baseline, the cells with significantly higher copy numbers were the EBV-predominant cell types.

### Statistical analysis

Survival was calculated from the date of diagnosis until the date of death from any cause or the date of the last follow-up. Progression-free survival (PFS) was calculated from the usage of PD-1 inhibitors to either progression or death from any cause. All statistical analyses of the data were performed using GraphPad Prism (version 9.0) and SPSS (version 20.0) statistical software. The Fisher’s exact test was used to analyze the association of molecular and laboratory parameters with treatment response. A P value < 0.05 were considered significant.

## Results

### Patient characteristics

A total of 16 CAEBV patients were enrolled in this study, with the clinical and hematological characteristics given in **Table 1**. Among the 16 patients, 12 were men and 4 were women, with a median age at onset of 33 years (range, 11-67 years) (**Table 1**). Before PD-1 inhibitors, all patients exhibited typical signs of CAEBV, including fever (13/16), lymphadenopathy (5/16), splenomegaly (9/16), hepatomegaly (4/16), cytopenia (8/16), abnormal liver function (8/16), and elevated serum ferritin levels (8/16). Two patients were diagnosed with hydroa vacciniforme, while 14 patients were diagnosed with sCAEBV. EBV-DNA copies were elevated in PB of all 16 patients (range, 9.15*102 –1.85*107 copies/ml), and plasma EBV-DNA loads ranged from 5.00*102 –1.76*105 copies/ml. The target cells of infection in 12 CAEBV patients were NK cells and 4 were T cells.

**Table 1:** Clinical and laboratory features of patients before PD-1 inhibitor infusion.

Patient	involving cells	Clinical features	WBC, ×10^9^/L	PLT, ×10^9^/L	ALT, U/L	AST, U/L	LDH, U/L	Ferritin, ng/mL	PBMC EBV-DNA, copies/mL	Plasma EBV-DNA, copies/ml
1	NK	Fever	2.46	38	59	56	511	2877.7	2.31E+05	1.38E+03
2	NK	Fever	3.69	78	63	114	219	105.8	1.85E+07	1.76E+05
3	NK	Fever, lymphadenectasis	4.48	239	39	68	272	539.8	1.02E+04	5.00E+02
4	T	Fever, lymphadenectasis, splenomegaly, hepatomegaly	2.65	146	23	28	267	25.5	4.85E+04	5.00E+02
5	T	Fever, hydroavacciniforme	3.64	281	17	23	240	28.4	1.75E+06	6.89E+03
6	T	Fever, rash, melena, splenomegaly, hepatomegaly	2.13	160	16	34	222	32.7	6.35E+05	5.00E+02
7	NK	Anemia, rash, hepatic dysfunction, splenomegaly, hepatomegaly	5.60	307	73	51	393	71.7	1.00E+07	2.08E+03
8	NK	Lymphadenectasis	31.13	230	29	17	175	356.4	9.15E+02	3.48E+03
9	NK	Fever, pancytopenia, splenomegaly, hepatomegaly	0.39	21	43	113	860	999.1	1.00E+05	1.90E+04
10	NK	Fever, diarrhea, splenomegaly	3.36	170	18	19	184	222.0	8.80E+05	5.00E+02
11	NK	Fever, lymphadenectasis, splenomegaly, hydroavacciniforme	7.75	162	181	36	250	311.3	1.54E+06	8.57E+03
12	NK	Fever, dental ulcer	4.52	163	44	38	375	411.7	3.28E+06	1.05E+03
13	NK	Fever	1.98	91	31	18	394	773.8	1.02E+06	5.24E+03
14	NK	Fever, splenomegaly	1.37	59	31	30	365	1264.5	7.81E+05	6.61E+03
15	T	Fever, lymphadenectasis, splenomegaly	5.30	285	19	20	178	541.6	9.99E+05	5.00E+02
16	NK	Splenomegaly, hepatic dysfunction	3.10	126	475	223	397	932.2	5.70E+06	8.80E+02

WBC, white blood cell; PLT, platelet; ALT, alanine aminotransferase; AST, aspartate aminotransferase; LDH, lactate dehydrogenase; PBMC, peripheral blood mononuclear cell; EBV, Epstein-Barr virus.

**Figure 1:**
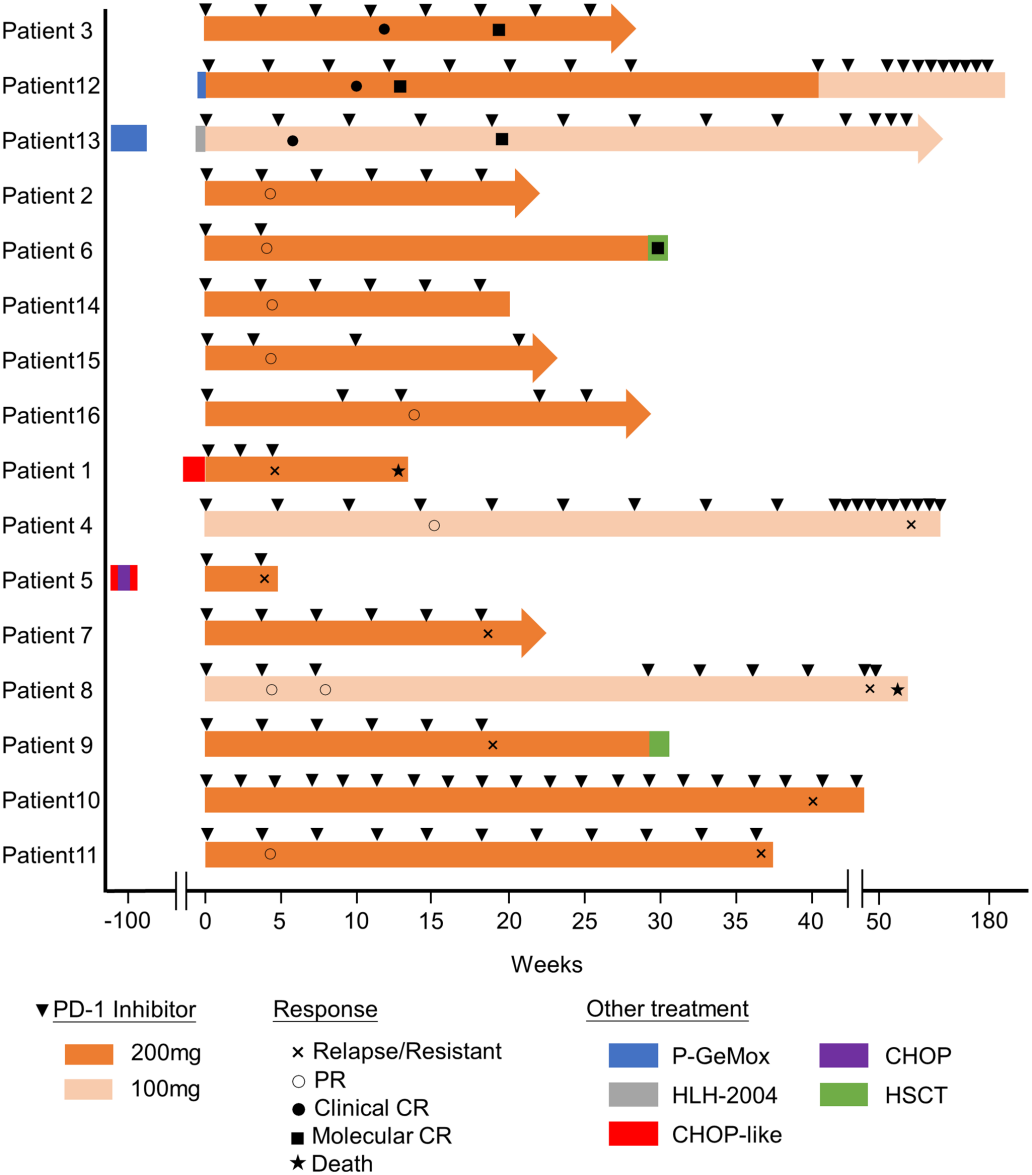
Swimmer plot of time on treatment of 16 CAEBV patients. P-GEMOX, pegaspargase, gemcitabine and oxaliplatin; CHOP, cyclophosphamide, doxorubicin, vincristine and prednisolone; HLH-2004, etoposide and dexamethasone.

Before PD-1 inhibitors therapy, 12 patients had only received antiviral drugs and/or corticosteroids, and 4 patients had received chemotherapy. They were treated with CHOP (cyclophosphamide, doxorubicin, vincristine and prednisolone) or CHOP-like regimens, P-GEMOX (pegaspargase, gemcitabine and oxaliplatin) and HLH-2004 regimens (**Figure 1**). Three patients were poorly controlled by chemotherapy, while one patient developed CAEBV recurrence after short-lived improvement. The median time from the diagnosis of CAEBV to PD-1 inhibitor therapy was two months (range, 0.2-80.2 months). Patients received PD-1 inhibitor therapy every 3 to 4 weeks, 100 to 200 mg intravenously, including pembrolizumab (2/16), sintilimab (9/16) or nivolumab (5/16) (**Table 2**).

**Table 2:** Treatments and outcomes of PD-1 inhibitor infusions.

Patient	Dose(cycles)	Outcomes	Survival(months)	PFS(months)
1	Pembrolizumab 100mg (3)	NR, DOD	3.8	1.9
2	Sintilimab 200mg (6)	PR	40.8+	8.8+
3	Sintilimab 100mg (19)	cCR and mCR	11.5+	5.5+
4	Pembrolizumab 200mg (8)	PR, NR	42.1+	18.2
5	Sintilimab 200mg (2)	NR	86.7+	5.1
6	Nivolumab 200mg (2)	PR	79.5+	53.6+
7	Nivolumab 200mg (6)	NR	57.7+	55.8+
8	Nivolumab 100mg (9)	PR, NR, DOD	15	13.4
9	Sintilimab 200mg (6)	PR, NR	7.7+	5.8
10	Sintilimab 200mg (20)	NR	13.8+	5.2
11	Sintilimab 200mg (11)	PR, NR	9.8+	8.1
12	Nivolumab 200mg (8), 100mg (12)	cCR and mCR	51.5+	49.5+
13	Sintilimab 100mg (13)	cCR and mCR	46.8+	15.7+
14	Nivolumab 200mg (6)	PR	55+	54.8+
15	Sintilimab 200mg (4)	PR	7.4+	4.9+
16	Sintilimab 200mg (5)	PR	7.1+	6.9+

PFS, progression-free survival; cCR, clinical complete response; mCR, molecular complete response; PR, partial response; NR, no response; DOD, dead of disease.

### Safety

Persistent or intermittent fever was the most common adverse event after the first infusion of PD-1 inhibitors. Four patients developed high fever, which was alleviated after symptomatic treatment such as antipyretic. No immune-related adverse events have been observed except Patient 14 suffered immune-related pancreatitis after 6 cycles of PD-1 inhibitor therapy, which was considered by physicians to be possibly related to PD-1 inhibitors. Therefore, the patient was asked to stop the PD-1 inhibitor and switch to other treatment plans.

### Efficacy

**Figure 2:**
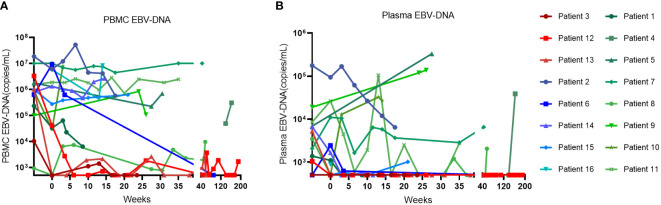
The change of EBV-DNA copy numbers after PD-1 inhibitor infusions in patients. **(A)** EBV-DNA copy numbers in the peripheral blood of patients. **(B)** EBV-DNA copy numbers in the plasma of patients.

The swimmer plot of time on treatment of 16 CAEBV patients was summarized in **Figure 1**. Among the 16 patients, 12 patients responded to PD-1 inhibitors, the median duration of PFS was 11.1 months (range, 4.9-54.8 months) (**Table 2**). Three of them achieved clinical CR, as well as molecular CR, the median time and cycles from the first application of PD-1 inhibitor to clinical CR were 6 weeks (range, 4-10 weeks) and 3 cycles (range, 2-4 cycles). During treatment, EBV-DNA copy numbers in the plasma and PB were gradually decreased in 3 patients (**Figure 2**), and molecular CR was achieved after a median of 16.7 weeks (range, 6.1-18.4 weeks) and 5 cycles (range, 3-6 cycles) of PD-1 inhibitor infusion. Nine patients achieved PR. Of the 9 patients who achieved PR, five patients continued treatment with PD-1 inhibitors and the median duration of PR was 8.8 months (range, 3.8-54.8 months), while four patients converted to NR. Two patients later underwent hematopoietic stem cell transplantation after stopping PD-1 inhibitors therapy and achieved CR without severe acute graft-versus-host disease.

### Comprehensive characterization of responders and non-responders

PD-L1 expression was evaluated in 3 patients treated with PD-1 inhibitors. Although the numbers were small, the immunohistochemical analysis showed that patients with high PD-L1 expression in tumor tissue may have a more significant response to PD-1 inhibitors: patients 3 and 12 who achieved CR showed higher PD-L1 scores, while patient 11 with negative PD-L1 expression did not respond to PD-1 inhibitors. Although the course of HV may be very indolent, the patients with HV have lower response to PD-1 inhibitors. Two patients were diagnosed with HV: patient 11 did not respond to PD-1 treatment, and patient 5 progressed after five-months improvement.

To explore the prognostic factors in CAEBV patients treated with PD-1 inhibitors, we analyze correlations with response to PD-1 inhibitors and the common clinical and laboratory features (**Supplemental Table 1**). However, a correlation of treatment outcome was not observed with blood count, AST level, ALT level, LDH level, cytokine level (IL6, IL8, IL2R) or ferritin level (**Supplemental Table 1**).

**Figure 3:**
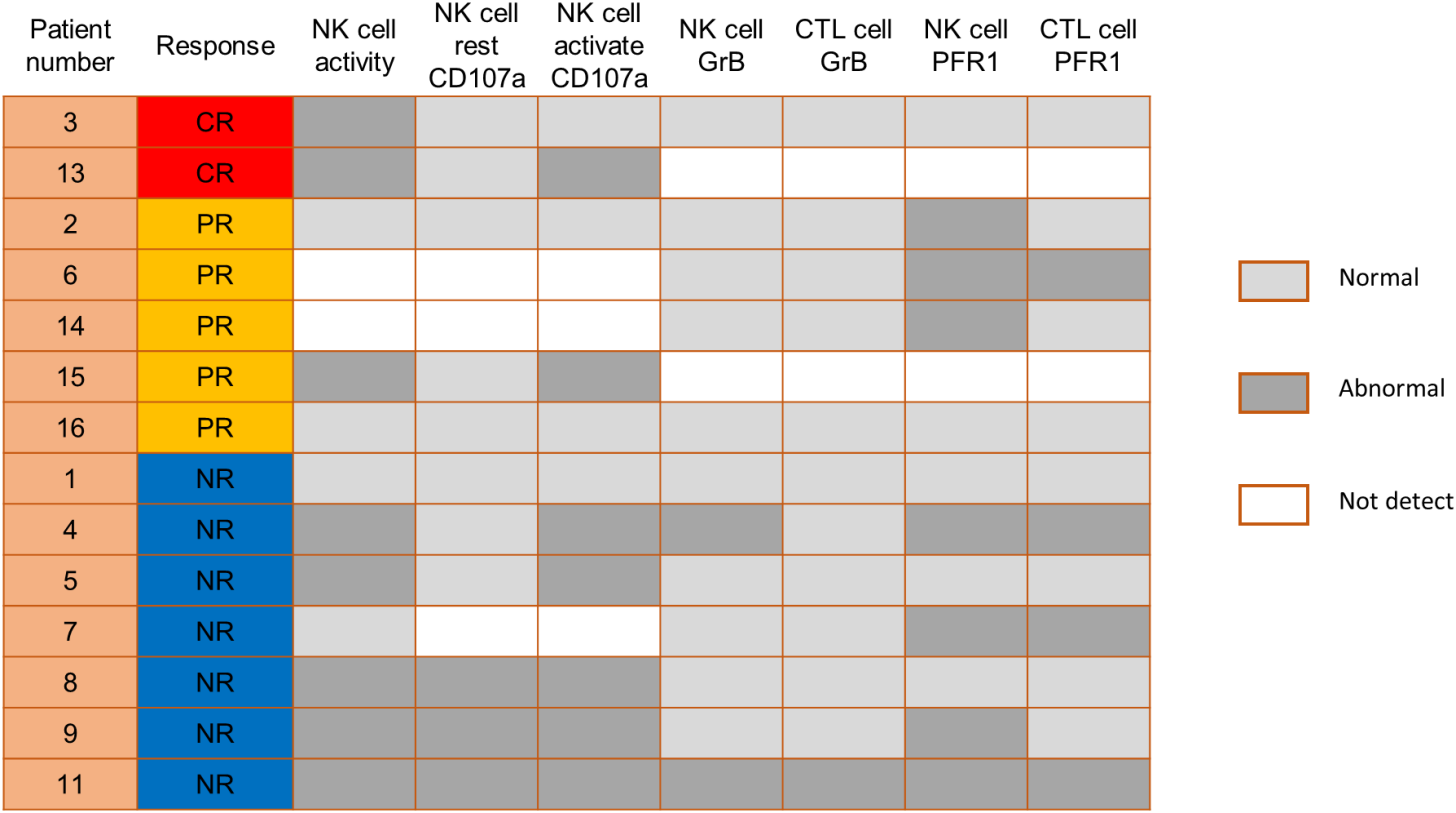
Comparison of NK cell function before PD-1 inhibitor infusion according to responses. CTL, cytotoxic T cell; GrB, granzyme B; PFR1, perforin 1.

**Figure 4:**
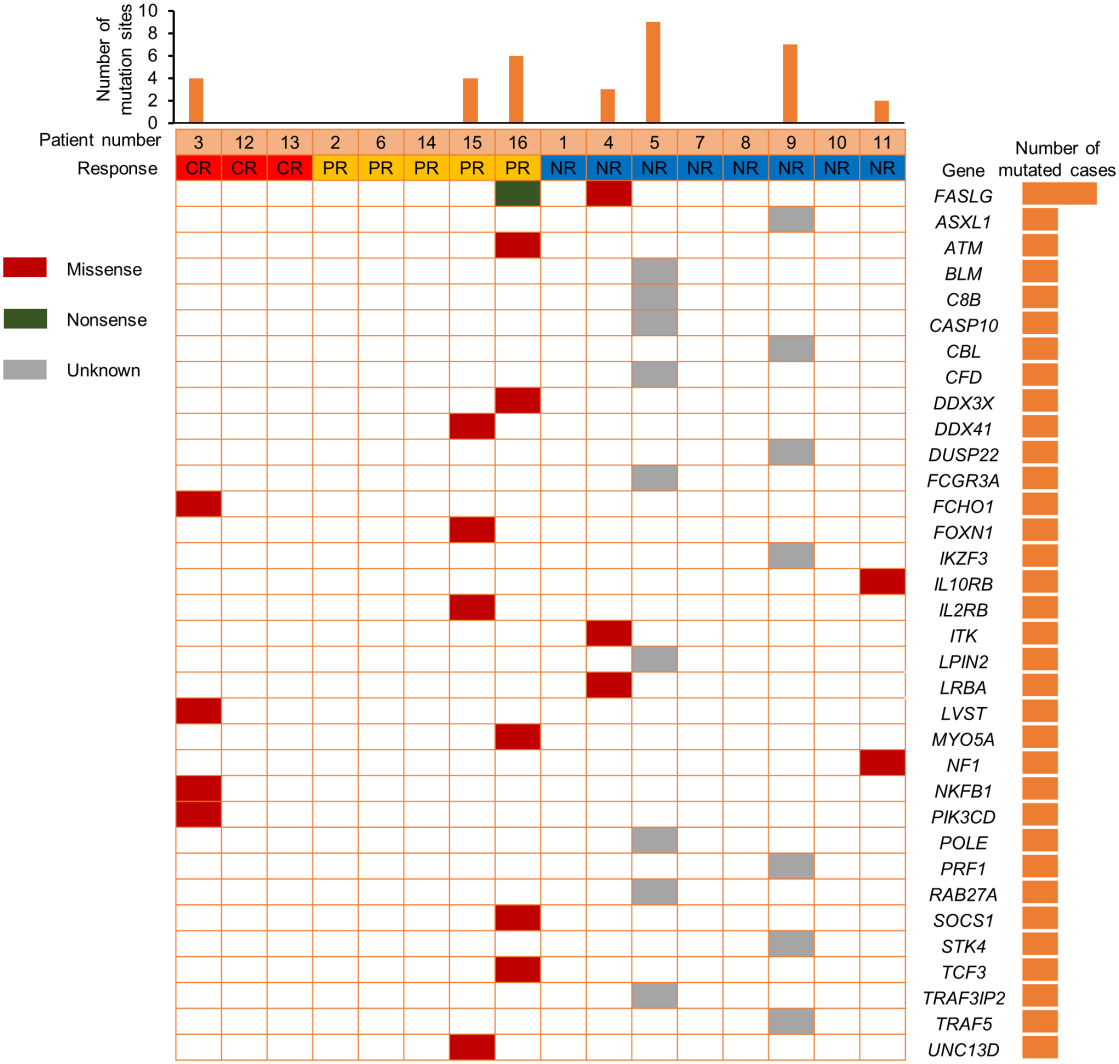
Comparison of mutation profiles according to responses.

We also compared the NK cell activation and degranulation function of responders and non-responders by expression of CD107a, granzyme B and perforin 1. The test results showed higher numbers of patients with defective NK cell function among non-responders (**Figure 3**). Genetic testing indicated that, except for 1 patient without gene sequencing and 8 patients with no related gene mutation detected, the other 7 patients were detected with potentially pathogenic or susceptibility mutations (**Figure 4**). Although our analysis failed to observe genes with high mutation rates, more gene mutations were observed in NR patients.

## Discussion

EBV is the first confirmed human tumor virus.1,2 CAEBV is now regarded as EBV+T/NK-LPDs EBV is the first confirmed human tumor virus (1, 2). CAEBV is now regarded as EBV+T/NK-LPDs according to the most recent WHO classification of tumors of hematopoietic and lymphoid tissues. Some patients with CAEBV can rapidly progress to fatal outcomes due to life-threatening complications, including HLH, multi-organ failure, and progression to T or NK cell malignant lymphomas such as ENKTL and aggressive NK cell leukemia (ANKL) (6). Although various therapies have been used to relieve the symptoms, only HSCT is regarded as the only curative therapy (6). However, the clinical application of allo-HSCT is limited by the high incidence of transplant-related complications and the availability of matched donors. Therefore, there is an urgent need to develop an effective better treatment.

In NK/T-cell lymphomas, EBV exists in a latency phase II state and expresses immunogenic antigens LMP1 (6). which can overexpress PD-L1 in EBV-infected lymphoma cells by acting on the enhancer and promoter of the PD-L1 gene (15, 25). Binding of PD-L1 on lymphoma cells to PD-1 on cytotoxic cells inhibits T cell cytotoxicity, leading to immune escape of lymphoma cells (26). Inhibition of the interaction between PD-1 and PD-L1 reverses EBV or cancer-related immunosuppression, which will restore the immunity needed to suppress cancer cells and clear EBV infection. PD-1 inhibitors have been shown to be effective against EBV+ gastric cancer (18), refractory/relapse ENKTL (19–21) and refractory/relapse EBV-HLH (22). Therefore, it is of great significance to explore the role of PD-1 inhibitors regimen in CAEBV treatment. Here, this study is the first retrospective series of CAEBV patients without HLH using PD-1 inhibitors.

In this retrospective study, we found out that PD-1 inhibitor has a significant effect on controlling disease and eradicating EBV in CAEBV patients. Among the 16 CAEBV patients without HLH, 12 patients responded to PD-1 inhibitors. Three of them achieved a sustained CR with remission of all features of the disease and no recurrence, and five patients achieved sustained PR. During treatment, EBV-DNA copy numbers in the plasma and PB were gradually decreased in 3 CR patients. The safety and tolerability of PD-1 inhibitors were acceptable, and no drug-related severe adverse effects were observed. One patient who suffered immune-related pancreatitis was recovery after withdrawal of PD-1 inhibitor and treatment of glucocorticoids.

Remarkably, the median time and cycles from the first application of PD-1 inhibitor to clinical CR in 3 patients with clinical CR were 6 weeks (range, 4-10 weeks) and 3 cycles (range, 2-4 cycles). These results suggest that PD-1 inhibitor has a rapid onset of action in patients who can achieve CR. Therefore, based on our limited experience, for CAEBV patients with PD-1 inhibitors, we suggest that efficacy evaluation should be conducted after 3 cycles of medication, and then patients who fail to achieve clinical CR can consider HSCT or combining therapy with other drugs on time. Although prior PD-1 inhibitor might affect the outcome of subsequent allo-HSCT (27), two patients who underwent HSCT did not develop severe acute graft-versus-host disease, indicating that PD-1 blockade may be used as a bridge to allo-HSCT in patients with CAEBV. On the other hand, PD-1 inhibitor combined with other drugs has shown benefits in some tumors with higher response rates (28, 29), which is an option worth considering for CAEBV and needs further studies.

According to the final results of this study, PD-1 inhibitors can achieve sustained control of CAEBV with tolerable toxicity. However, regrettably, some patients did not obtain any clinical benefit (4/16) or relapsed after a brief remission (4/16). Therefore, selecting CAEBV patients with a high probability of responding to PD-1 inhibitors is crucial to improve the outcome of PD-1 inhibitors. A comprehensive analysis of responders showed that the response to PD-1 inhibitor was not associated with clinical parameters such as blood count, AST level, ALT level, LDH level, cytokine level (IL6, IL8, IL2R) or ferritin level.

It has been reported that PD-L1 overexpression was suggested as a potential biomarker for PD-1/PD-L1 targeting therapy in some solid malignancies (30) and PD-L1 is highly expressed in EBV-LPD pathological tissues (15–17), but previous studies showed whether PD-L1 expression could be a potential biomarker to predict response to PD-1/PD-L1 inhibitors are still largely undefined in relapsed/refractory NK/T-cell lymphoma (19, 21, 31, 32). In this study, PD-L1 expression was evaluated in 3 patients treated with PD-1 inhibitors: 2 patients who achieved CR showed higher PD-L1 scores, while another patient with negative PD-L1 expression did not respond to PD-1 inhibitors. The result suggested that the role of PD-L1 expression in PD-1/PD-L1 blockade needs to be further explored in CAEBV patients. In this study, we also analyzed the classification of CAEBV because a previous study showed that HV had a better prognosis and more favorable course than sCAEBV in CAEBV patients (33). However, the patients with HV in our study have lower response to PD-1 inhibitor. Considering the retrospective analysis and small numbers of this study, this result needs to be validated further.

In addition, previous research by Hsu et al. suggested that NK cells are potential responders to PD-1/PD-L1 blockade and the efficacy of PD-1/PD-L1 blockade in some tumor mouse models depends on NK cell activity (34). They showed upregulation of PD-1 expression on NK cells in the tumor microenvironment, which effectively inhibits NK cell-mediated tumor immunity. In our study, comparing the NK cell function of responders and non-responders, higher numbers of patients with defective NK cell function among non-responders. This is consistent with the published reports that NK cells also mediate the effect of PD-1/PD-L1 targeting therapy (34). Therefore, we speculate that the assessment of NK cell function might help identify patients more likely to respond to PD-1 treatment. Genetic testing indicated that 7 patients were detected with potentially pathogenic or susceptibility mutations, and most of the gene mutations detected may be associated with immunodeficiency and tumorigenesis. Although our analysis failed to observe single genetic defect with high mutation rates in responders and non-responders, more gene mutations were observed in NR patients.

Therefore, among CAEBV patients receiving PD-1 inhibitors, those with strong NK cell activity, positive PD-L1 expression and no gene mutations were more likely to respond. Furthermore, because of the small number of cases, there are some limitations in interpreting the efficacy of PD-1 inhibitors based on our results. In the future, larger, systematically protocol-driven prospective, randomized controlled clinical trials and further mechanism studies are still needed to clarify the actual role of PD-1 inhibitors in CAEBV and its underlying mechanisms.

In summary, PD-1 inhibitors showed good activity in a subset of CAEBV patients without HLH. The assessment of PD-L1 expression, NK cell function and gene mutation might be helpful for identifying patients more likely to respond to this drug. In addition, patients who fail to achieve clinical CR after 3 cycles of PD-1 inhibitors can consider HSCT or combining therapy with other drugs promptly. However, more studies focusing on CAEBV patients with PD-1 inhibitors are needed.

## Data availability statement

The original contributions presented in the study are included in the article/**Supplementary Material**. Further inquiries can be directed to the corresponding author.

## Ethics statement

The studies involving human participants were reviewed and approved by the Medical Ethics Committee of the Department of Hematology, Tongji Hospital, Tongji Medical College, Huazhong University of Science and Technology. Written informed consent to participate in this study was provided by the participants’ legal guardian/next of kin.

## Author contributions

MZ and LH designed and supervised the clinical study. YB, HL and JW collected and analyzed the clinical data. YM and PZ performed statistical analyses. YM and PZ and MZ drafted and revised the manuscript. All authors contributed to the article and approved the submitted version.
