# Supplementary material for: Outcomes of programmed death protein-1 inhibitors treatment of chronic active Epstein Barr virus infection: A single center retrospective analysis
Source: Front Immunol. 2023 Mar 10;14:1093719. doi: 10.3389/fimmu.2023.1093719 (PMC10036359; doi:10.3389/fimmu.2023.1093719)
Supplement: Supplementary file 1 [file Table_1.docx]

| **Characteristics** | **Total, n** | **Responders, n** | **Nonresponders, n** | ***P* value** |
| --- | --- | --- | --- | --- |
| **ALT** |  |  |  | 0.358 |
| Normal | 9 | 4 | 5 |  |
| Increased | 7 | 5 | 2 |  |
| **AST** |  |  |  | 0.633 |
| Normal | 10 | 5 | 5 |  |
| Increased | 6 | 4 | 2 |  |
| **Ferritin** |  |  |  | 0.315 |
| Normal | 8 | 6 | 2 |  |
| Increased | 8 | 3 | 5 |  |
| **IL2R** |  |  |  | 1.000 |
| Normal | 8 | 4 | 4 |  |
| Increased | 8 | 5 | 3 |  |
| **IL6** |  |  |  | 1.000 |
| Normal | 5 | 2 | 3 |  |
| Increased | 11 | 7 | 4 |  |
| **IL8** |  |  |  | 1.000 |
| Normal | 10 | 6 | 4 |  |
| Increased | 6 | 3 | 3 |  |
| **LDH** |  |  |  | 0.596 |
| Normal | 5 | 2 | 3 |  |
| Increased | 11 | 7 | 4 |  |
| **PLT** |  |  |  | 0.596 |
| Normal | 11 | 7 | 4 |  |
| Decreased | 5 | 2 | 3 |  |
| **WBC** |  |  |  | 1.000 |
| Normal | 8 | 4 | 4 |  |
| Decreased | 8 | 5 | 3 |  |

**Supplemental table 1** Characteristics of patients before treatment.
